# Acupressune and Its Effects

**Published:** 1868-10

**Authors:** 


### Acupressune and its Effects.

Dr. Hutchinson presented to the New York Pathological Society, January 22, 1868, some specimens illustrating the effects of acupressure on the arteries of sheep, dogs, and men, and remarked that "these specimens, in company with those presented at a previous meeting, comprise my experience with acupressure.—It has been employed in twenty-eight arteries in man and five or six in the lower animals. Among other operations in which it was employed were, two amputations of the leg, one at knee-joint and one of foot, and one of wound in the radial artery; which latter, by the way, illustrated the value of acupressure in a striking manner. The patient had suffered from an extensive lacerated wound in the lower part of the forearm and hand, and, several days after, sloughing took place followed by hemorrhage from the radial artery. The bleeding was very profuse, so much so that the patient lost a pint and a half of blood in a very few minutes. The artery was exposed in the midst of sloughing tissue. The tourniquet had been applied and also the persulphate of iron; these were, however, removed and acupressure needles slipped under the artery above and below, with the effect of arresting the hemorrhage in an instant. The needles were removed at the end of twenty-two hours. The following day the patient had hemorrhage from the superficialis volæ from the same cause. The blood oozed out very freely, and a considerable quantity was lost before the house-surgeon could arrive to arrest it. This Dr. Eldridge did by the method of acupressure with a very satisfactory result. This latter needle was removed at the end of twenty-seven hours. These two cases illustrate very strongly the value of acupressure in certain cases. I believe that if a ligature had been applied premature sloughing might have resulted with its attendant hemorrhage. In either case, in order to prevent this occurrence, it would have been necessary to have applied a ligature in sound tissure above and below the wound, to have made a very long, tedious, and unsatisfactory dissection, with the result, as before stated, of having secondary hemorrhage afterwards.

"The value of acupressure is, of course, not fully known. Yet it has been demonstrated that we can by these means certainly arrest hemorrhage. I am disposed to think that secondary hemorrhage is less apt to occur after it than after ligature. It is very well known that secondary hemorrhage, from some peculiarity of the constitution, does not form a firm enough clot before the ligature separates. Then, again, it occurs as the result of ulcerations of the arteries prematurely and from sloughing, which not infrequently extends beyond the seat of the plug. By means of acupressure we simply compress the coats of the artery together; no violence is used, the coats are not disturbed at all, and there is on that account less liability to secondary hemorrhage."—*Medical Record.—Am. Jour. Med. Sciences.*

